# The Microtubule Minus-End Binding Protein Patronin Is Required for the Epithelial Remodeling in the *Drosophila* Abdomen

**DOI:** 10.3389/fcell.2021.682083

**Published:** 2021-07-21

**Authors:** Sadhana Panzade, Maja Matis

**Affiliations:** ^1^Interfaculty Centre ‘Cells in Motion,’ University of Münster, Münster, Germany; ^2^Institute of Cell Biology, Medical Faculty, University of Münster, Münster, Germany

**Keywords:** microtubules, Patronin, *Drosophila* abdomen, cell migration, tissue morphogenesis

## Abstract

In the developing *Drosophila* abdomen, the epithelial tissue displays extensive cytoskeletal remodeling. In stark contrast to the spatio-temporal control of the actin cytoskeleton, the regulation of microtubule architecture during epithelial morphogenesis has remained opaque. In particular, its role in cell motility remains unclear. Here, we show that minus-end binding protein Patronin is required for organizing microtubule arrays in histoblast cells that form the *Drosophila* abdomen. Loss of Patronin results in a dorsal cleft, indicating the compromised function of histoblasts. We further show that Patronin is polarized in these cells and is required for the formation of highly dynamic non-centrosomal microtubules in the migrating histoblasts. Thus, our study demonstrates that regulation of microtubule cytoskeleton through Patronin mediates epithelium remodeling.

## Introduction

Tissue remodeling results from coordinated cell behavior such as proliferation, migration, cell intercalation, apoptosis, extrusion, and remodeling of cell-cell contacts. In *Drosophila*, the abdominal epidermis forms by extensive remodeling of histoblasts, diploid imaginal cells specified during embryogenesis (Guerra et al., 1973). Four distinct pairs of histoblast nests give rise to the adult abdominal segments (Mandaravally Madhavan and Schneiderman, 1977). During the larval stages, the histoblasts remain dormant and only begin proliferating during early metamorphosis induced by the hormone Ecdysone (Roseland and Schneiderman, 1979). In the first phase of abdominal development, they proliferate and spread without replacing the larval epidermal cells (LECs). Following first rapid cell divisions at around 15 h after puparium formation (APF), anterior and dorsal histoblasts nests fuse before the onset of migration. Dorsal histoblasts continue to divide further and migrate (Supplementary Video 1), upon which they finally fuse at the dorsal midline at around 36 h APF (Madhavan and Madhavan, 1980; Ninov et al., 2007, 2010; Bischoff and Cseresnyes, 2009). During this phase of histoblasts migration, LECs extrude and get engulfed by hemocytes (Ninov et al., 2007). Over the last decade, the importance of the cytoskeleton in tissue remodeling has been shown for many tissues (Heisenberg and Bellaiche, 2013; Ladoux and Mege, 2017). The picture that has emerged from these studies is that actin, together with myosin, forms contractile arrays that are key constituents of different morphogenetic processes ranging from epithelial folding to cell intercalation and tissue convergence (Pollard and Cooper, 2009; Lecuit et al., 2011; Heisenberg and Bellaiche, 2013; Pinheiro and Bellaiche, 2018). However, while these studies have established the importance of actin-based mechanical forces, relatively little is known about the contribution of other cytoskeletal components, such as microtubules, to different cell behaviors during morphogenesis (Matis, 2020).

Microtubule filaments are composed of alpha (α) and beta (β) tubulin heterodimers that assemble into proto-filaments to form hollow tubes. They exhibit constant growth and shrinkage events, which are regulated by interaction with distinct microtubule-associated proteins and motors. The microtubule plus ends showcase fast dynamics of growth and shrinkage, whereas the minus-ends exhibit *in vitro* two to three times slower dynamics of growth and shrinkage phases than the plus ends (Desai and Mitchison, 1997). In cells, microtubule plus ends are responsible for the growth of the microtubules and dynamic interactions with different subcellular compartments, especially at the cell cortex (Noordstra and Akhmanova, 2017). In contrast, the minus-ends determine the organization of microtubule networks due to the nucleation or stabilization at the specific subcellular sites (Wu and Akhmanova, 2017). Their highly dynamic nature is fundamental for the reorganization of the microtubule cytoskeleton, and allows adaptation to the diverse repertoire of function that they play in cells. Microtubules participate in cell division by facilitating the segregation of chromosomes, provide tracks for the trafficking of cargos, and are essential in maintaining the cellular shapes, cell scaffolding and during polarized cell migration (Bouchet and Akhmanova, 2017; Forth and Kapoor, 2017; Muroyama and Lechler, 2017; Singh et al., 2018; Burute and Kapitein, 2019). Hence, they are involved in various functions required during tissue morphogenesis (Matis, 2020; Roper, 2020).

Microtubule formation requires interaction with proteins that nucleate/stabilize microtubule minus-end (Akhmanova and Hoogenraad, 2015). The major nucleation factor that directly associates with minus-ends and facilitates the growth of microtubules is the gamma-tubulin ring complex (γ-TuRC) (Kollman et al., 2011). In addition, there are other known proteins that specifically target free microtubule minus-ends, like spindle associated abnormal spindle microencephaly-associated protein (ASPM) or acylation-stimulating-protein (Asp) in *Drosophila*, the KAT8 regulatory NSL complex subunit (KANSL) and plant-specific microtubule-associated protein 2 (SPIRAL2) (Akhmanova and Steinmetz, 2019). Finally, members of the calmodulin regulated spectrin associated protein (CAMSAP) family in vertebrates and Patronin in invertebrates were recently identified to recognize microtubules at the minus-end independent of γ-TuRC (Hendershott and Vale, 2014).

*Drosophila* Patronin, or short spindle protein 4 (Ssp4), was the first protein identified in the CAMSAP/Patronin family (Goodwin and Vale, 2010). Patronin and CAMSAPs are essential for the stabilization and formation of non-centrosomal microtubules by protecting the minus-end from depolymerizing effects of the kinesin-13 family depolymerases [e.g., kinesin-like protein at 10 A (Klp10 A)] (Hendershott and Vale, 2014). The minus-end of microtubules is recognized by the C-terminal carboxy-terminal domain (CKK), a highly conserved sequence across diverse eukaryotes (Atherton et al., 2017, 2019). Recently, it was shown that the CAMSAP/Patronin family plays an essential role in organizing/patterning microtubule cytoskeleton in several differentiated cells, which have enriched non-centrosomal microtubules. During cell differentiation, when cells exit mitosis, the centrosome is often inactivated, and a new microtubule-organizing center is established. In general, the assembly of a new microtubule organization center (MTOC) involves the relocalization of γ-TuRC or CAMSAP/Patronin to the new site. In epithelial cells, non-centrosomal microtubules are organized along the apical-basal axis, with their microtubule minus-ends concentrated at the apical cortex, which acts as a MTOC. In *Drosophila* epithelium during dorsal fold formation and follicle cells, microtubule anchoring to the apical cortex depends on the Patronin (Khanal et al., 2016; Takeda et al., 2018). Similarly, mammalian CAMSAP3 plays an essential role in maintaining apicobasal oriented microtubules in mouse intestinal cells and human Caco-2 cells (Noordstra et al., 2016; Toya et al., 2016). In the *Drosophila* oocyte, Patronin, together with the spectraplakin Short stop (Shot), localizes to the apical membrane domain in the anterior part of the oocyte leading to the formation of anterior-posterior aligned microtubule arrays (Nashchekin et al., 2016). Another common site that serves as a microtubule-organizing center in several settings is the Golgi apparatus. Also, here, γ-TuRC and CAMSAP/Patronin family act as major microtubule anchoring factors. For example, in endothelial cells, CAMSAP2 promotes nucleation of microtubules at Golgi that are crucial for the formation of stable protrusions that enable directional migration on 2D substrates and in 3D soft matrices (Martin et al., 2018). Furthermore, CAMSAP/Patronin family was reported to be involved in the anchoring of microtubules at the nuclear surface (Zheng et al., 2020) and organizing non-centrosomal microtubules in neurons (Feng et al., 2019; Wang et al., 2019). Thus, the Patronin/CAMSAP proteins regulate microtubule organization in various differentiated cells. How the microtubule cytoskeleton is coordinated during these morphogenetic processes, however, is not known.

Here we set out to probe the role of minus-end binding protein Patronin in regulating microtubule dynamics during epithelial morphogenesis. Consistent with a function of Patronin in the polarization of the microtubule network, we find that temporal downregulation of Patronin disrupts normal abdominal development. Furthermore, the quantitative analysis of microtubule cytoskeleton revealed a loss of microtubules and switch from non-centrosomal microtubule organization to centrosomal organization. Finally, the disappearance of cortical microtubules correlates with slower and less directional histoblast migration and leads to aberrant dorsal abdomen formation. Collectively, these results show for the first time that Patronin coordinates cell behavior during extensive epithelial remodeling of the *Drosophila* abdomen.

## Materials and Methods

### Fly Genetics and Stocks

All flies used in experiments were reared and maintained at 25°C on cornmeal fly food with dark/light cycle. For most experiments, white pre-pupae (0 h APF) of the desired genotype were collected and staged at 25°C. Following fly lines that are described in FlyBase and were obtained from the Bloomington Drosophila stock center (BDSC), the Vienna *Drosophila* Resource Center (VDRC), or the Kyoto *Drosophila* Genetic and Genomic Resources Center (DGGR), unless noted otherwise were used: *UAS-EB1-GFP (BDSC 35512), Ecad-mTomato (BDSC 58789), UAS-Tub-EOS (BDSC 51313), Arm-GFP (BDSC 8555), tubGal4 (BDSC 5138), UAS-Lifeact-GFP (BDSC 35544)*, *FRT42DtubGal80 (II), FRT42Dsspey^05252^ (DGRC 114436), hsFLP, Tub-Gal4 (BDSC 5138), UAS-GFP (BDSC 5430) sqh-EYFP-Golgi (BDSC 7193), His2avD-mRFP (BDSC 23651), Patronin* RNAi (*VDRC108927*), *Ubi-beta-TubGFP (DGRC 109604)*, *UAS-palmKate2-K7 (attP2) (III) from Stefan Luschnig* (*University of Münster*, *Germany*), *GFP-Patronin22H2-N (attP40) (III)* (Takeda et al., 2018), *ciGal4* (Croker et al., 2006), and *Ubi-p63E-cnn-RFP* (Conduit et al., 2010).

For clonal analysis *patronin*^*ey05252*^ FRT42D mutant positively marked clones were generated using FLP-mediated recombination. Clones were induced by heat-shock of prepua (0 h APF) at 37°C for 10 min in a water bath.

### Dissection

Pupae for imaging were transferred on the glass slide coated with double tape and placed on their dorsal side. Using forceps and scissors, the whole pupal case was removed. The pupae were transferred on a coverslip smeared with glue diluted in heptane solution.

### Image Acquisition and Processing

The images were acquired in the apical plane of the epithelial cells along with z-stacks of 7 μm with a step size of 1.5 or 0.5 μm using a Zeiss 710 confocal microscope. The third (A3) abdominal segment was analyzed due to the flat tissue in the middle region that allowed to image morphogenesis of the abdomen over many hours. All images and movies were processed with the Fiji/ImageJ software (NiH, version 2.0.0.) Images/movies of collectively migrating epithelial cells were aligned along the dorsal axis using Fiji (the transform and rotate tool). For microtubule plus-tip tracking, single cells from the entire collectively migrating sheet of epithelium were cropped using a cell outline marker.

### Microtubule Plus-Tip Tracking and Analyses

Movies for microtubule +TIP analysis were acquired using an upright Zeiss AxioImager M2 microscope with a Yokogawa CSU10B spinning disk, an sCMOS ORCA Flash 4.0LT system, and a Plan-Apochromat 100×/1.46 (NA) oil immersion objective. To visualize microtubule dynamics, we used microtubule plus-end binding protein EB1-GFP. To mark the cell boundaries for analysis and to image only in the apical planes, cells were labeled with the adherens junction marker E-cad-mTomato. Images were taken in 300–500 ms intervals with 488 and 561 nm wavelength lasers and the first 75 frames were analyzed using the MATLAB-based open-source software u-track 2.2.0 (Applegate et al., 2011). Parameters used for tracking EB1 comets are listed in Supplementary Table 1. The cell elongation index was calculated by measuring the minimum value divided by the maximum value of Feret diameters using the ROI function in Fiji.

Subpopulations of microtubules were obtained by dividing the total microtubule population based on mean growth speed and mean growth lifetime (see Supplementary Tables 3, 4). Based on this, we identified four clusters, namely [fast short-lived], [fast long-lived], [slow short-lived], and [slow long-lived]. For all four subpopulations, the mean percentage was compared between wild type and patronin mutant cells.

### Cell Nuclei Tracking and Analyses

To track epithelial cells during migration toward the dorsal midline, we used a nuclear marker H2AV-mRFP (Histone-mRFP). Movies were acquired during developmental stages 24–29 h APF when cells are actively migrating and processed using Fiji software. For migration, cell nuclei were tracked for at least 50 min in 60 min long movies using the tracking algorithm autoregressive motion. A maximum distance to allow the spots/objects to deviate from the predicted position, which accounts for any directional changes during tracking, was specified in IMARIS software. From the analysis, we extracted nuclei tracks, their length, duration, speeds, and straightness indices. The straightness index is calculated based on the following formula in IMARIS:

$$\mathrm{The}\,\mathrm{straightness}\,\mathrm{index}=\frac{\mathrm{Track}\,\mathrm{Displacement}}{\mathrm{Track}\,\mathrm{Length}}$$

The track displacement is the distance between the first and the last position:

$$\text{Track}\,\text{displacement}=\sqrt{{\text{D}}_{\text{x}}\,{\left(\mathrm{tL},\mathrm{tF}\right)}^{2}+{\text{D}}_{\text{y}}\,{\left(\mathrm{tL},\mathrm{tF}\right)}^{2}+{\text{D}}_{\text{z}}\,{\left(\mathrm{tL},\mathrm{tF}\right)}^{2}}$$

$$D_{x}\,\left(t_{1},t_{2}\right)=P_{x}\,\left(t_{1}\right)-P_{x}\,\left(t_{2}\right)$$

where *D* = track displacement, *tL* = last time index of track, *tF* = first time index of track, and *P*_*x*_(*t*) = *x*-position of object at time index *t.*

The track length is the total length of displacements within the track:

$$\text{Track length =}\sum_{t=t\,F\,1}^{t\,L}\sqrt{D_{x}\,{\left(t,t-1\right)}^{2}+D_{y}\,{\left(t,t-1\right)}^{2}+D_{z}\,{\left(t,t-1\right)}^{2}}$$

$$D_{x}\,\left(t_{1},t_{2}\right)=P_{x}\,\left(t_{1}\right)-P_{x}\,\left(t_{2}\right)$$

where *L* = track length, *tL* = last time index of track, *tF* = first time index of track, and *P*_*x*_(*t*) = *x*-position of object at time index *t.*

### Fluorescence Intensity Analyses

To quantify Patronin polarity, all images were aligned along the axis of migration. The line was drawn using the ROI function in Fiji from the back of the cell to the front. The PlotProfile feature in Fiji was used to obtain the pixel intensities along the line for E-cadherin and Patronin signals in the cell. The intensities along the long axis of each cell were normalized to the cell length of 1, wherein 0 indicates the back and 1 indicates the front of the cell. The observations are grouped in 10 quantiles by normalized location. To test whether the difference is significant, the observations are divided into five quantiles and regression analysis is performed for Patronin fluorescence intensity on dummy variables corresponding to five groups of the longitudinal axis of the cell location, where the second group (0.4 < = *x* < 0.6) is the baseline. The individual cell fixed effects are included to account for the possible heterogeneity in fluorescence intensity levels across cells. The standard errors are clustered at the cell level to account for potential variation in the error term (i.e., residual variance across them). All data were analyzed using R software version 4.0.3.

### Cell Division Analysis

To quantify the number of cell divisions during collective cell migration, the divisions were counted in movies taken between 26 and 30 h APF. The area was calculated for the whole image using the area selection tool in Fiji.

### Statistical Analyses

All graphs and statistics were performed with Graphpad PRISM software version 8 and R software version 4.0.3. All data were tested for normal distribution using the D’Agostino–Pearson normality test. Statistical significance was calculated using Student’s *t*-test (two-tailed) and a non-parametric Mann–Whitney *U*-test for datasets with non-normal distributions, as indicated in the corresponding figure legends. A Kruskal–Wallis test was used for multiple comparisons. n.s indicates non-significance. The significance codes are ^∗^*p* ≤ 0.05, ^∗∗^*p* ≤ 0.01, ^∗∗∗^*p* ≤ 0.001, and ^****^p ≤ 0.0001.

## Results

### Patronin Is Required for Abdominal Morphogenesis

**FIGURE 1:**
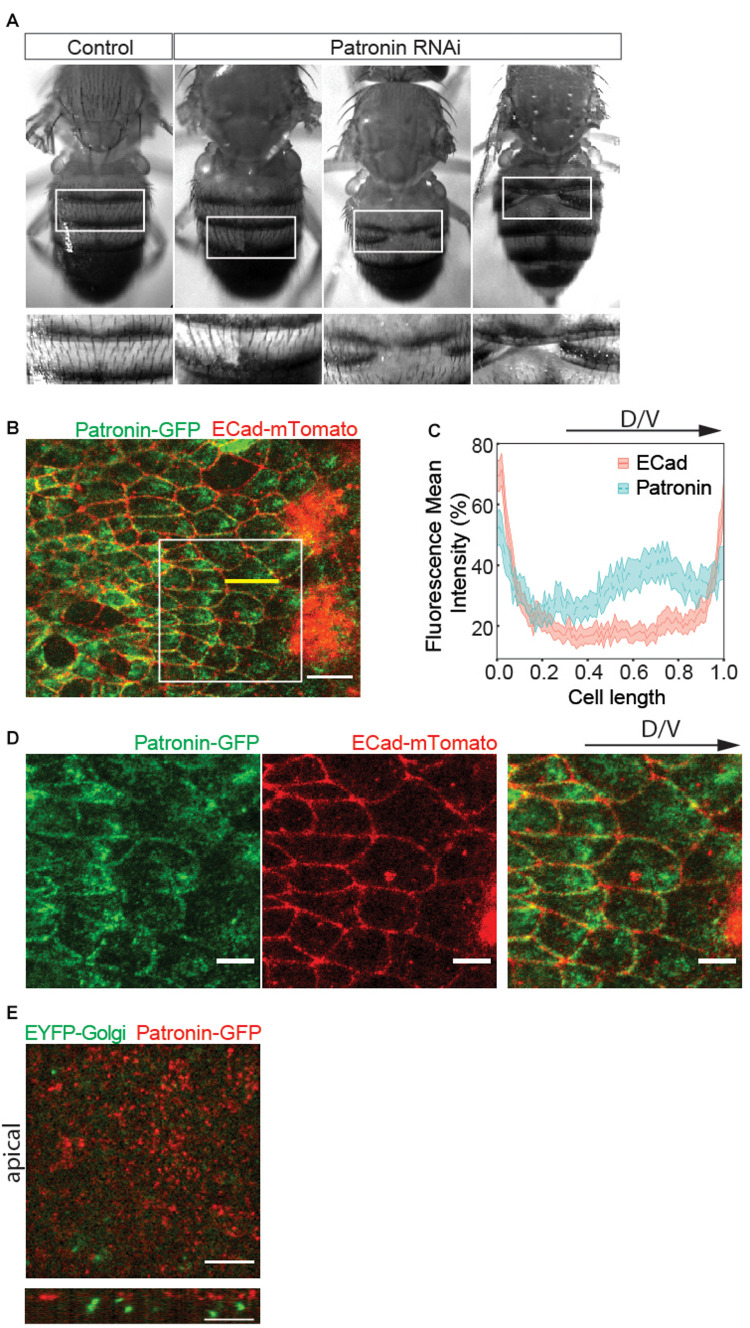
Patronin is required for abdominal morphogenesis. **(A)** Adult abdomens overexpressing *patronin* RNAi exhibit clefts. Insets: closeups of adult abdomens in boxed regions. The number of affected individuals in control (*Patronin* RNAi *VDRC108927* line only) was 0/116 adults and in overexpressing *patronin* RNAi was 13/100 adults. Unpaired *t*-test Welch’s correction: *p* = 0.0083 (**) for overexpressing *patronin* RNAi compared to the control. **(B)** A representative live fluorescence image of 27–28 h after puparium formation (APF) abdomen showing histoblast expressing Patronin-GFP (green) and the adherens junction marker ECad-mTomato (red). Scale bar = 10 μm. **(C)** Quantification of fluorescence intensities of Ecad-mTomato (red) and Patronin-GFP (green) signals along the longitudinal axis of cells. The intensities along the long axis of each cell [shown with a yellow line in panel **(B)**] were normalized to the cell length of 1 and averaged. The regression analysis includes cell fixed effects and standard errors are clustered by cell (see Supplementary Tables 1A,B). The shaded area around the mean indicates the 95% confidence interval. Number of cells (*n*) = 92 and pupae (*N*) = 3. **(D)** Insets from panel **(B)** showing Patronin-GFP (green, left), ECad-mTomato (red, middle), and merged (right). Scale bar = 5 μm. **(E)** Live fluorescence images of EYFP-Golgi marker and Patronin-RFP. Top view (above) and cross-section (below). Developmental stage **(B–E)**: 27–28 h APF. Scale bar, 5 μm.

To probe for the potential role of Patronin during morphogenesis, we performed RNAi mediated knockdown of Patronin using the *cubitus interruptus* (*ci*) Gal4 driver, which is expressed in the anterior segments. A small number of adult animals (13%) exhibited dorsal closure phenotype (Figure 1A). This abnormal epidermis development is the first indicator for a Patronin-dependent defect during dorsal tissue remodeling.

Abdominal epithelial morphogenesis relies on a well-characterized sequence of events (Ninov et al., 2007, 2010). Here, cells specified during embryogenesis first proliferate and subsequently migrate to replace LECs (Supplementary Video 1). To investigate the role of Patronin in this process, we analyzed its localization in migrating histoblast. Strikingly, live-cell imaging of endogenously tagged Patronin (EGFP-Patronin) (Takeda et al., 2018) showed enrichment at cell junctions as well as at the cell front in the apical plane (Figures 1B,D,E). To quantify the subcellular localization of Patronin, we compared the fluorescence intensities of Patronin and E-cadherin along the longitudinal axis of the cell in the apical plane. We find Patronin enrichment toward the front of the cells compared to E-cadherin (Figure 1C and Supplementary Tables 1A,B). A previous study demonstrated that the mammalian homolog of Patronin, CAMSAP2, co-localizes with the front positioning Golgi in migrating endothelial cells (Martin et al., 2018). Interestedly, our data revealed that in histoblasts, Patronin associated with the apical cortex and did not co-localize with the Golgi marker, which is positioned basally (Figure 1E). Thus, our *in vivo* results indicate that the apical accumulation of Patronin in motile epithelial cells is reminiscent of non-motile epithelial (Nashchekin et al., 2016; Noordstra et al., 2016; Toya et al., 2016; Takeda et al., 2018) rather than motile endothelial cells (Martin et al., 2018).

### Microtubule Organization and Dynamics in Migrating Histoblasts

Microtubules play a pivotal role in cell migration by providing tracks for intracellular transport, supporting cell mechanics and controlling signaling. In general, migrating cells have a dense network of centrosomal and non-centrosomal microtubules that are polarized along the front-rare axis (Etienne-Manneville, 2013). However, it was previously reported that some cell types in the *Drosophila* embryo lack functional centrosome, and with this also centrosomal microtubules, during interphase (Rogers et al., 2008). As Patronin/CAMSAP family binds to microtubule minus-ends, which enables the formation of non-centrosomal microtubules, we hypothesized that Patronin is part of a non-centrosomal microtubule organization center (ncMTOCs), and its accumulation at the cell front may be required for proper microtubule organization during cell migration.

**FIGURE 2:**
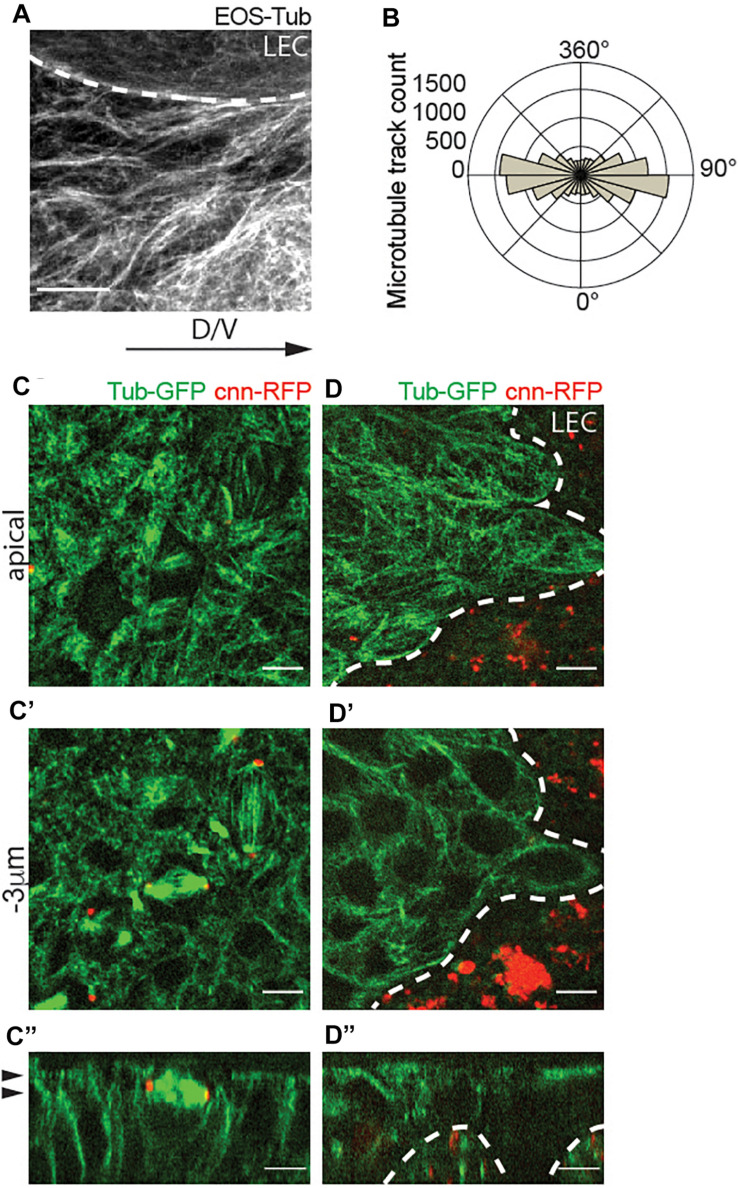
Apical non-centrosomal microtubules in histoblast are aligned along the axis of migration (dorso-ventral axis). **(A)** Live fluorescence images of microtubules in wild-type histoblasts labeled with Tub-EOS. Scale bar = 5 μm. **(B)** Microtubule growth track polarity in a cell. Polar plots indicate the counts (frequency of microtubule tracks) of microtubules growing in a cell along the longer axis of the cell. Number of microtubules (*n*) = 13,208, cells (*n*) = 28, and pupae (*N*) = 3. **(C–D”)** Confocal sections of abdomen showing histoblast expressing Tub-GFP (green) and the centrosome marker Cnn-RFP (red) during the initial phase of spreading **(C–C”)** and later active migration phase **(D–D”)**. Panels are showing the apical cross-sections **(C,D)**, cross-section 3 μm bellow the apical side **(C’,D’)**, and orthogonal views of the histoblasts **(C”,D”)**. A white dashed line is marking the border between histoblasts and larval epidermal cells (LECs) **(A,D–D”)**, and the black arrowheads are marking the position of the cross-section plane in [top in panels **(C,D)**, and bottom in panels **(C’,D’)**]. Note that interphase histoblasts do not have functional centrosomes. Scale bar **(C–D”)**, 5 μm.

To test our hypothesis, we characterized the spatial organization of microtubules in histoblasts. In these cells, microtubules visualized by EOS-α-Tubulin (EOS-Tub) were abundant in the apical plane at the level of adherens junctions, which are organized horizontally along the apical cortex (Figure 2A). Angular distribution of microtubules showed alignment along the dorsal-ventral direction throughout the migration phase (Figure 2B). To investigate the centrosome function during the cell cycle in more detail, we co-expressed the RFP tagged centrosomal marker Centrosomin (Cnn-RFP) and β-tubulin-GFP (Tub-GFP). We find that during mitosis, Cnn assembled into a dense structure marking the centrosome, but vanished during mitotic exit and was not visible during the interphase (Figures 2C–D”). On contrary, the LECs which are polyploid contain multiple copies of active microtubule-nucleating centrosomes during interphase (Figures 2D–D”). This result establishes that interphase histoblasts do not contain a centrosome with constitutive MTOC activity, but completely switch to non-centrosomal microtubules.

**FIGURE 3:**
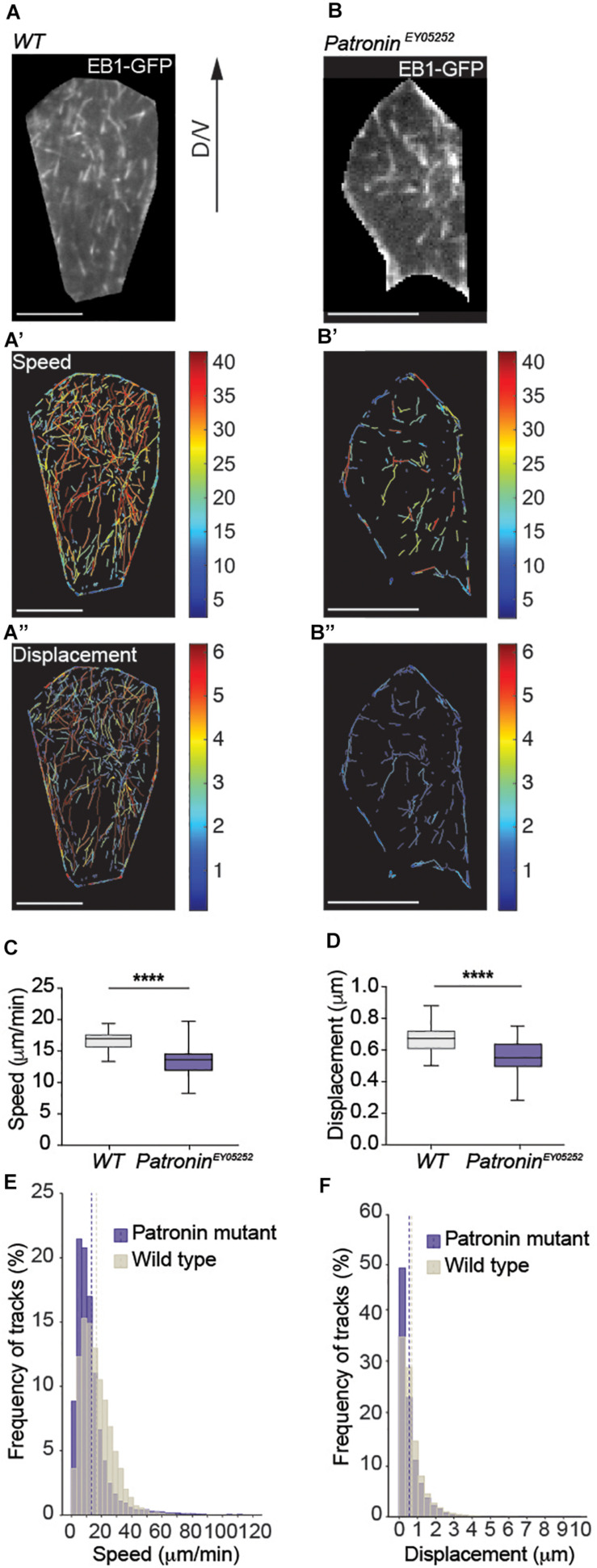
Dynamics of non-centrosomal microtubules in histoblast. **(A)** Live fluorescence images of EB1-GFP in wild-type histoblasts and corresponding color-coded tracks for growth rate **(A’)** and persistent growth/displacement **(A”)** as indicated. Scale bars, 20 μm. **(C,D)** Quantification of growth speeds and persistent growth for microtubules in wild-type and *patronin*^*ey05252*^ mutant histoblasts. Graph shows mean growth speeds calculated for cells. Unpaired *t*-test Welch’s correction, *p* < 0.0001 (****) for **(C,D)**, number of microtubules (*n*) = 13,208, cells (*n*) = 28, and pupae *N* = 3 for wild-type and number of microtubules (*n*) = 9,184, cells (*n*) = 53, and pupae (*N*) = 11 for *patronin*^*ey05252*^ mutant cells. **(E,F)** Histograms of growth speeds and persistent growth for all microtubules. Number of microtubules (*n*) = 13,208, cells (*n*) = 28, and pupae *N* = 3 for wild-type and number of microtubules (*n*) = 9,184, cells (*n*) = 53, and pupae (*N*) = 11 for *patronin*^*ey05252*^ mutant cells. The dashed line shows mean growth speed and mean growth displacement for wild-type and *patronin*^*ey05252*^ mutant cells. Graphs in panels **(C,D)** show box plots with a line indicating the mean and maximum and minimum values. Scale bar, 5 μm.

To substantiate this finding, we analyzed the relationship between MTOC and microtubule outgrow by tracking plus-end binding protein EB1 tagged with GFP (EB1-GFP) that displays characteristic EB1 comets (Figure 3A). Interestingly, microtubules were emanating from many discrete sites in the cytoplasm and junctions and did not exhibit a classical single origin of nucleation that leads into the astral organization of microtubules (Supplementary Video 2 and Supplementary Figures 1A,A’). However, we could see multiple EB1 outgrowths from the same spot, what could be explained by the formation of multiple small astral-like networks at apical Patronin foci (Figure 1D).

**FIGURE 4:**
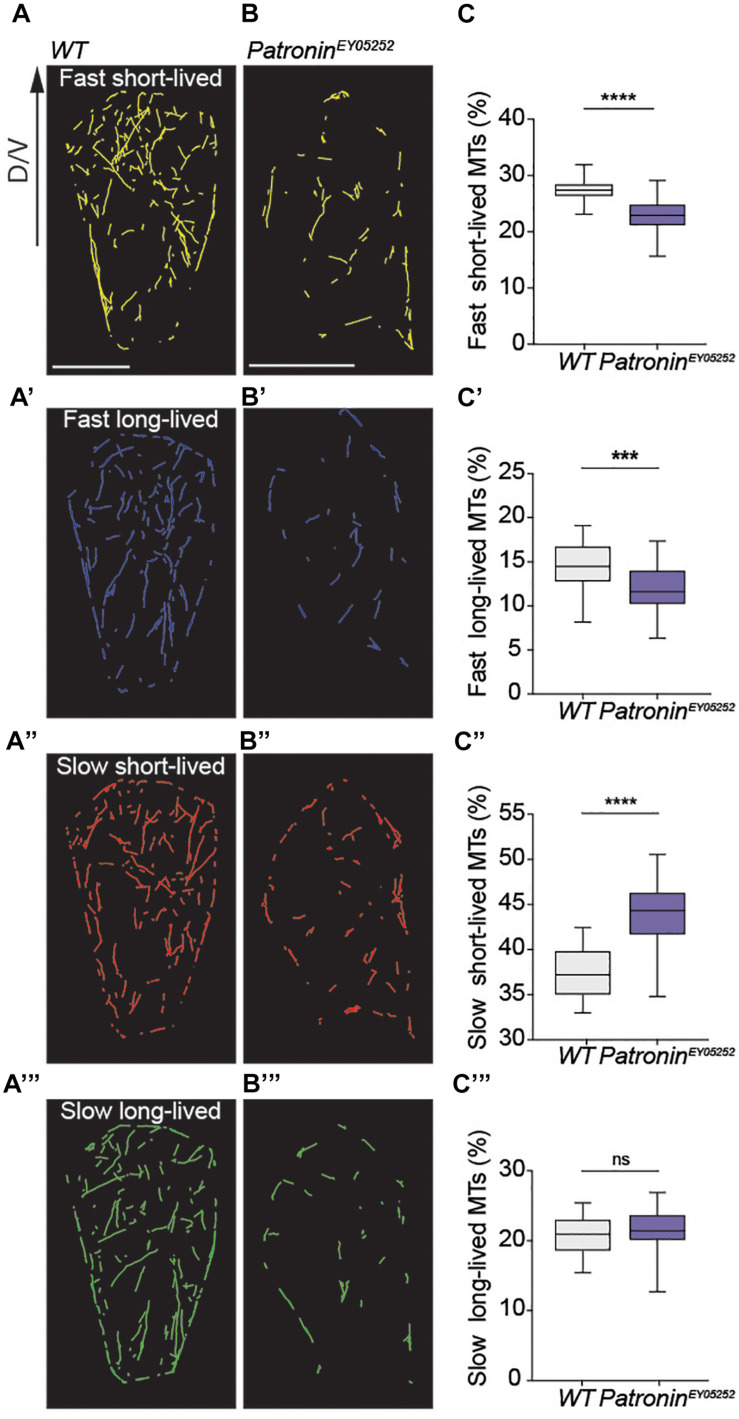
Loss of Patronin leads to the reduction of the dynamic microtubules. **(A–C”’)** Proportions of microtubule subpopulation calculated based on mean growth speed and lifetime. **(A–B”’)** Sub-tracks for four distinct dynamic subpopulations of growing microtubules in a cell as, fast and short-lived (yellow), fast and long-lived (blue), slow and short-lived (red), and slow and long-lived (green) for a representative wild-type **(A–A”’)** and *patronin*^*ey05252*^ mutant cell **(B–B”’)**. **(C–C”’)** Quantification of four distinct dynamic subpopulations in wild-type and *patronin*^*ey05252*^ mutant histoblasts. Box plots show the percentage subpopulation for microtubules clustered based on growth speed and lifetime mean values. Graphs show box plots with a line indicating the mean and with maximum and minimum values. In all plots: ns, no significant difference; *p* < 0.001 (***); *p* < 0.0001 (****); and unpaired *t*-test Welch’s correction, number of microtubules (*n*) = 13,208), cells (*n*) = 28, and pupae (*N*) = 3 for wild-type and number of microtubules (*n*) = 9,184, cells (*n*) = 53, and pupae (*N*) = 11 for *patronin*^*ey05252*^ mutant cells. Scale bar **(A–B”)**, 5 μm.

Our data to this point revealed that migrating histoblasts rely on microtubules that are entirely independent on a central MTOC. In a next step, we analyzed the dynamics of non-centrosomal microtubules during migration. The quantification of microtubule growth revealed a mean growth speed of 16.81 μm/min and a mean persistent growth length of 0.68 μm (Figures 3A’,A”,C–F and Supplementary Tables 2–6). These parameters are consistent with the microtubule plus-end dynamics in other cells (Zwetsloot et al., 2018). To directly test for a potential causal link between Patronin localization and microtubule organization and dynamics, we generated homozygous MARCM clones of the *patronin*^*ey05252*^ allele and analyzed the microtubule organization. Strikingly, loss of Patronin led to a transformation of the non-centrosomal microtubule array that is present in wild-type cells (Figure 3A and Supplementary Video 2) into a more radial pattern, whereby the majority of the microtubules converged in one spot with occasionally observing additional microtubule growth from other sites (Figure 3B, Supplementary Video 3, and Supplementary Figures 1B,B’). Next, we quantified the nucleation events to assess the effect on microtubule numbers. Notably, the number of nucleation events was reduced by around 65% (Supplementary Tables 3, 4), demonstrating that Patronin is required to protect or stabilize microtubule minus-ends. Moreover, the microtubule dynamics in *patronin*^*ey05252*^ mutant histoblast was also changed, with a mean growth speed of 13.35 μm/min and a mean persistent growth length of 0.56 μm (Figures 3B’,B”,C–F). To probe for distinct subpopulations of microtubules within cells, we clustered the data into four groups based on the mean growth speed and growth lifetime (Supplementary Figures 2A,B and Supplementary Tables 3, 4). Using this distinction, we then scored subcellular microtubule subpopulations. Interestingly, dynamic microtubules represented the majority of microtubules in wild-type cells (Figures 4A-A”’, C-C”’). In contrast, *patronin*^*ey05252*^ mutant cells displayed a loss of the fast-growing microtubules, both short- and long-lived were reduced compared to the control, while the percentage of the slow growing microtubules was elevated (Figures 4B–C”’). The observed reduction in microtubule dynamics suggests changes in spatial organization of the cytoskeleton. In wild-type cells, microtubules grow along the cell cortex, where many regulators of the microtubule dynamics are localized (Etienne-Manneville, 2013; Mayor and Etienne-Manneville, 2016; Noordstra and Akhmanova, 2017). In *patronin*^*ey05252*^ mutant histoblasts, a lower number of microtubules that are more randomly distributed within the cell contacts less frequently the cell cortex and thus could be less affected by cortically localized microtubule regulators.

Together, our analysis revealed that interphase histoblasts do not have active centrosomes to support the astral organization of the microtubule network typical for many cell types. Thus, these findings indicate an essential and very specific role of Patronin in supporting the formation of dynamic non-centrosomal microtubule networks during epithelium remodeling.

### Histoblast Migration Requires Patronin Function

**FIGURE 5:**
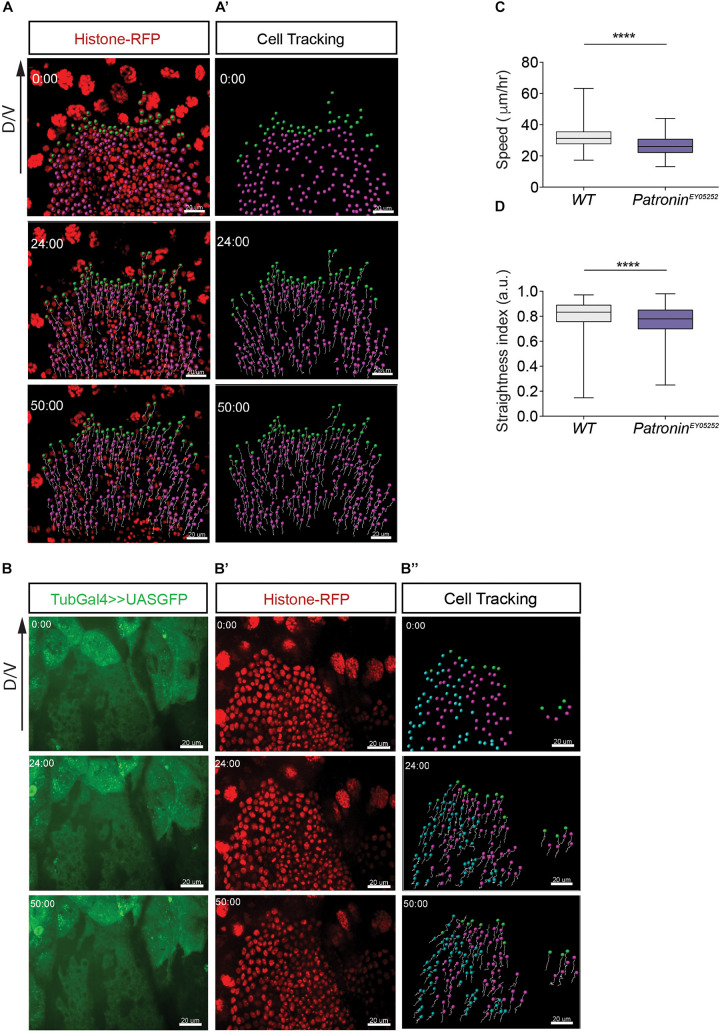
Histoblast lacking Patronin show migration defects. **(A)** Representative images of migrating wild-type histoblasts in segment A3 expressing nuclear marker Histone-mRFP. **(A’)** The tracks are representative paths of the cells moving in the direction of migration axis. **(B)** Representative images of migrating *patronin*^*ey05252*^ mutant histoblasts in segment A3 labeled with GFP and expressing nuclear marker Histone-mRFP **(B’)**. **(B”)** The tracks are representative paths of the wild-type cells (blue), *patronin*^*ey05252*^ mutant cells (magenta) and leading cells (green) moving in the direction of migration axis. The panels **(A–B”)** represent snapshots of histoblast from 0 to 50 min during cell migration. Scale bars, 20 μm. **(C)** Quantification of speeds for wild-type and *patronin*^*ey05252*^ mutant histoblasts. Mann–Whitney *U*-tests, *p* < 0.0001 (****), number of cells (*n*) = 1,649, and pupae (*N*) = 3 for wild-type cells and number of cells (*n*) = 1,139 and pupae (*N*) = 5 for *patronin*^*ey05252*^ mutant tissue. **(D)** The straightness index indicates the persistence of migration for wild-type and *patronin*^*ey05252*^ mutant histoblasts. Mann–Whitney *U*-tests, *p* < 0.0001 (****), number of cells (*n*) = 1,649, and pupae (*N*) = 3 for wild-type and number of cells (*n*) = 1,139 and pupae (*N*) = 5 for *patronin*^*ey05252*^ mutant tissue. Graphs in panels **(C,D)** show box plots with a line indicating the mean and maximum and minimum values. Developmental stage **(B–C)**: 24–29 h APF. Scale bars, 10 μm.

To further explore the requirement of Patronin in collective migration, we quantified the speed and persistence of migrating cells during the active phase of pupal development (24–29 h APF) by tracking cell nuclei labeled with RFP tagged Histone (Figures 5A–A’, B–B” and Supplementary Videos 4, 5). Compared to control cells, migration speed and directionality were both significantly reduced in *patronin*^*ey05252*^ mutant tissue (Figures 5C,D and Supplementary Figures 3A–B”), suggesting that the phenotype in adult flies lacking Patronin could be a consequence of defective migration during abdomen formation. Previous studies in S2 cells showed that Patronin localizes to centrosomes during mitosis, and its depletion results in a short spindle phenotype (Goodwin and Vale, 2010). To exclude the possibility that the observed defects in adult flies originate from perturbation of cell divisions, we determine if depletion of Patronin affects the cell cycle. A quantitative analysis of cell proliferation showed no differences in number of cell divisions between wild-type and in *patronin*^*ey05252*^ mutant tissues (Supplementary Figure 3C).

One characteristic of collective migration is that cells form a clear migrating sheet with the leaders at the edge and followers behind (Rorth, 2009; Mayor and Etienne-Manneville, 2016). The tracking of individual cells within sheets revealed that cells at the front and those behind it both contributed actively to the net movement (Rorth, 2009; Mayor and Etienne-Manneville, 2016). However, in many systems, leaders exhibit more motile and protrusive activity (Mayor and Etienne-Manneville, 2016). To gain deeper insight into the behavior of each cell type in the abdomen, we divided the histoblasts into two groups, leading and non-leading cells. During migration, both cell types in wild-type tissue maintained their speed and direction, indicating that they moved in a coordinated fashion toward the destined dorsal midline (Supplementary Figures 4A,B). One explanation for this lack of lateral or longitudinal exchange between cells is that leader histoblasts, unlike other leader cells in cohesive groups (e.g., epithelial cells during wound closure), do not have a contact-free edge and thus do not possess extreme front behavior. Instead, leading histoblasts make cell-cell contact with LEC. While these cells form protrusions (Ninov et al., 2007), we observed that protrusions of leading and non-leading cells do not differ (Supplementary Figure 5A). Moreover, histoblast protrusions, filopodia and lamellipodia, contained actin as well as microtubules (Supplementary Figures 5B,C). Collectively, these data reveal that cells in the *Drosophila* abdomen migrate as a cell sheet, whereby cells in the front, as well as followers, equally contribute to the collective movement.

Finally, we assessed the dynamics of microtubules in wild-type leader and follower cells. The analysis displayed a mild difference in the microtubule growth speed and displacement in leading and non-leading cells (Supplementary Figures 4C,D). Similarly, microtubules were aligned along the axis of migration in both cell types. This finding is not entirely surprising given that migration analysis showed no differences between these two types of cells (Supplementary Figures 4A,B). Thus, our results suggest that overall microtubule organization and dynamics are the same in the leading and non-leading cells and could contribute to the cell migration by enabling coordinated cell motility within an epithelial sheet.

## Discussion

Microtubules play a pivotal role in many cellular processes, and to be functional their organization has to be tunable to a variety of cell functions. During epithelial morphogenesis, they have to switch between centrosomal organization supporting cell division and non-centrosomal microtubules supporting the various functions of epithelial cells. The reorganization of the microtubule cytoskeleton depends on its ability to undergo dynamic instability, whereby they switch between growth and shrinkage phases. A variety of factors regulates microtubule dynamics and organization in order to facilitate proper cellular functions. In the *Drosophila* abdomen, the epithelial tissue extensively remodels to form the adult epidermis. This is a multistep process that involves division, migration, and fusion of histoblasts, which are coordinated with the migration and apoptosis of LECs (Ninov et al., 2007; Bischoff and Cseresnyes, 2009). It was previously shown that histoblast migration and LECs both require the actin cytoskeleton (Ninov et al., 2007). Complementing these studies, we find that microtubules are equally important for abdomen formation. Specifically, we identify Patronin, a microtubule minus-end binding protein, as an organizer of non-centrosomal microtubules in histoblast. Furthermore, we demonstrate that it is required for the collective cell migration and possibly other processes critical for the remodeling of the epithelium as a loss of the protein results in the aberrant abdomen formation.

In this study, we performed a detailed quantitative analysis of microtubule organization and dynamics during epithelial tissue morphogenesis. Given that Patronin binds to microtubule minus-ends, we propose that it is crucial for the organization of dynamic non-centrosomal microtubules. Consistently, our results revealed that histoblasts lose the active centrosome as they exit mitosis, hence interphase histoblasts contain only non-centrosomal microtubules. Moreover, Patronin foci in the abdominal epithelium are enriched at the apical cortex in a polarized manner. In line with this, we find that 60% of all microtubules (Figure 3E) in these cells are highly dynamic and concentrated at the apical cortex. Consistently, in *patronin* mutant cells microtubules not only changed from non-centrosomal to centrosomal organization but also displayed reduced dynamics. These findings are in line with the published role of microtubules controlling protrusion maintenance in migrating mesenchymal cells (Bouchet et al., 2016). We proposed that Patronin foci concentrated at the histoblast front are organizing non-centrosomal microtubules, which are supporting cell migration. Intriguingly, we find protrusions made by these cells to be enriched with microtubules. Furthermore, growing microtubule plus-ends are at the tip of filopodia (Supplementary Figure 5B). Thus, our findings suggest a conserved role for Patronin/CAMSAP family in cell migration *in vivo*.

We observed that the reduction of microtubules in *patronin*^*ey05252*^ mutant histoblasts is more severe than the observed defect in the cell migration. Although this hypomorphic allele reduces Patronin levels (Bellen et al., 2004), which decreases the density of the non-centrosomal microtubule network (Figure 3), the remaining microtubules could still be sufficient to rescue microtubule-dependent functions in cells (e.g., polarized transport, regulation of Rho GTPase signaling or mechanical support). In addition, multiple cell behaviors contribute to abdomen morphogenesis. These include, among others, histoblast proliferation, migration and fusion at the midline, as well as the active contribution of LECs to their replacement. Hence, the perturbed function of the microtubule cytoskeleton in histoblast is likely ameliorated by these co-occurring processes, manifesting in only weak migration defect. Consequently, the dorsal cleft phenotype seen upon knockdown of Patronin in the anterior segments of the abdomen is most likely due to perturbing cell migration and errors during the lateral fusion. Interestingly, during epithelial fusion, cellular protrusions are often present at the leading edges of the opposing tissues and provide the first contact between them (Pai et al., 2012; Begnaud et al., 2016). Moreover, it was proposed that microtubules are required for filopodia formation during the last step of dorsal closure in *Drosophila melanogaster* (Jankovics and Brunner, 2006). This is in line with our data showing that the leading edge of migrating histoblasts possess microtubule-containing filopodia. Unfortunately, we could not precisely analyze the migration pattern and filopodial dynamics during the fusion step because of the tissue bending. Future studies will be needed to investigate the role of Patronin in the regulation of filopodia during histoblast migration and fusion and will further clarify the role of microtubules for trafficking and force generation in these structures.

In summary, we demonstrate that the protein Patronin specifically stabilizes non-centrosomal microtubule minus-ends and plays an important role in abdominal epithelial remodeling. Mechanistically, Patronin organizes apical non-centrosomal microtubules, which correlates with the higher persistence and velocity of cell migration. As changes in microtubule organization are essential for many epithelial functions during morphogenesis, we anticipate that more cell processes that involve CAMSAP/Patronin family will be revealed in the future.

## Data Availability Statement

The original contributions presented in the study are included in the article/Supplementary Material, further inquiries can be directed to the corresponding author.

## Author Contributions

SP performed and analyzed the experiments, and wrote the manuscript. MM designed and supervised the research, and wrote the manuscript. Both authors contributed to the article and approved the submitted version.

## Conflict of Interest

The authors declare that the research was conducted in the absence of any commercial or financial relationships that could be construed as a potential conflict of interest.

## Supplementary Material

Supplementary Figure 1 Non-centrosomal microtubules in histoblast are aligned along the axis of migration (dorso-ventral axis). **(A,B)** A representative cell showing EB1 tagged with GFP in wild-type **(A)** and *patronin*^*ey05252*^ mutant histoblast **(B)**. **(A’,B’)** A maximum intensity projection of an EB1-EGFP time-lapse sequence. Developmental stage **(A–B’)**: 25–26 h APF. Scale bars, 5 μm.

Supplementary Figure 2 Analysis of four distinct dynamic subpopulations in wild-type and *patronin*^*ey05252*^ mutant histoblasts. **(A)** Quadrant scatter plot showing the classification of all detected microtubule growth tracks according to their growth speed and life time in the representative **(A)** wild-type cell (*n*) = 750 and **(B)** *patronin*^*ey05252*^ mutant cell (*n*) = 196. Each point represents one microtubule. Microtubule subpopulations are color coded as, fast and short-lived (yellow), fast and long-lived (blue), slow and short-lived (red), and slow and long-lived (green).

Supplementary Figure 3 Histoblast migration in *patronin*^*ey05252*^ mutant flies. **(A,B)** Live fluorescence images of migrating histoblast in segment A3. The *patronin*^*ey05252*^ mutant cells are labeled with the GFP **(A)** and the nuclei are labeled with the Histone-mRFP **(B)**. A yellow dashed line marks the border between histoblasts and LECs and a white dashed line marks the wild type cells. Panels **(A–B)** represent snapshots of histoblast from 0 to 59 min during cell migration. Scale bars **(A,B)**, 10 μm. **(C)** Quantification of the cell divisions per square micrometer in wild type cells and *patronin*^*ey05252*^ mutant cells. Bar plot is presented as mean ± SEM. Mann–Whitney *U* test, n.s., *p* = 0.6308, number of cell divisions (*n*) = 90, and pupae (*N*) = 3 for wild-type cells and number of cell divisions (*n*) = 146 and pupae (*N*) = 4 for *patronin*^*ey05252*^ mutant tissue.

Supplementary Figure 4 Leading and non-leading histoblast migrate same. Quantification of the speeds **(A)** and the straightness index **(B)** of leading (green) and non-leading epithelial cells (magenta). **(A,B)**. Bar plots are presented as mean ± SEM. Statistics **(A,B)**, Mann–Whitney *U* test, n.s., no significant difference; and *p* < 0.0001 (****). Number of leading cells (*n*) = 279, and pupae (*N*) = 3 and non-leading cells (*n*) = 1,392 and pupae (*N*) = 3. **(C, D)** Quantification of microtubule growth speed **(C)** and displacement length **(D)** in leading cells (green) and non-leading cells (magenta). Statistics **(C,D)**, Mann–Whitney *U* test (speed) *p* = 0.0241 and (displacement length) *p* < 0.0444. The number of leading cells for **(C,D)** (*n*) = 14 and pupae (*N*) = 3 and non-leading cells (*n*) = 14 and pupae (*N*) = 3. Developmental stage **(A–B)**: 26–30 h APF, **(C–D)**: 25–26 h APF.

Supplementary Figure 5 Histoblasts protrusions are enriched with actin and microtubules. **(A)** A representative confocal image showing migrating histoblast expressing membrane marker palm-mKate2. White arrowheads indicate protrusions in non-leading cells. **(B,C)** Cell protrusions in histoblasts are labeled with the membrane marker palm-mKate2 and microtubules with the plus-end marker EB1-GFP **(B)** and in panel **(C)** LifeAct-GFP marks actin. White arrowheads indicate the presence of microtubules **(B)** and actin **(C)** in protrusions. Developmental stage **(A–C)**: 25–26 h APF. Scale bars **(A–C)**, 5 μm.

Supplementary Video 1 Time-lapse video of migrating histoblast in segment A3 during dorsal abdomen formation (24–29 h APF) expressing Arm-GFP. Scale bar, 25 μm.

Supplementary Video 2 Time-lapse video of EB1-GFP marking microtubule plus-ends (25–26 h APF) in wild-type histoblast, related to Figure 3A. Scale bar, 5 μm.

Supplementary Video 3 Time-lapse video of EB1-GFP marking microtubule plus-ends (25–26 h APF) in *patronin*^*ey05252*^ mutant histoblast, related to Figure 3B. Scale bar, 5 μm.

Supplementary Video 4 Time-lapse video of migrating histoblast in segment A3. Histoblast nuclei are labeled with the Histone-mRFP and *patronin*^*ey05252*^ mutant cells are labeled with GFP, related to Supplementary Figure 3. Scale bar, 10 μm.

Supplementary Video 5 Time-lapse video of migrating histoblast in segment A3. The tracks represent paths of the cells expressing nuclear marker Histone-mRFP moving in the direction of migration axis, related to Figure 5. Scale bar = 20 μm.
