# Supplementary figures and images for: The Microtubule Minus-End Binding Protein Patronin Is Required for the Epithelial Remodeling in the Drosophila Abdomen
Source: Front Cell Dev Biol. 2021 Jul 21;9:682083. doi: 10.3389/fcell.2021.682083 (PMC8335404; doi:10.3389/fcell.2021.682083)

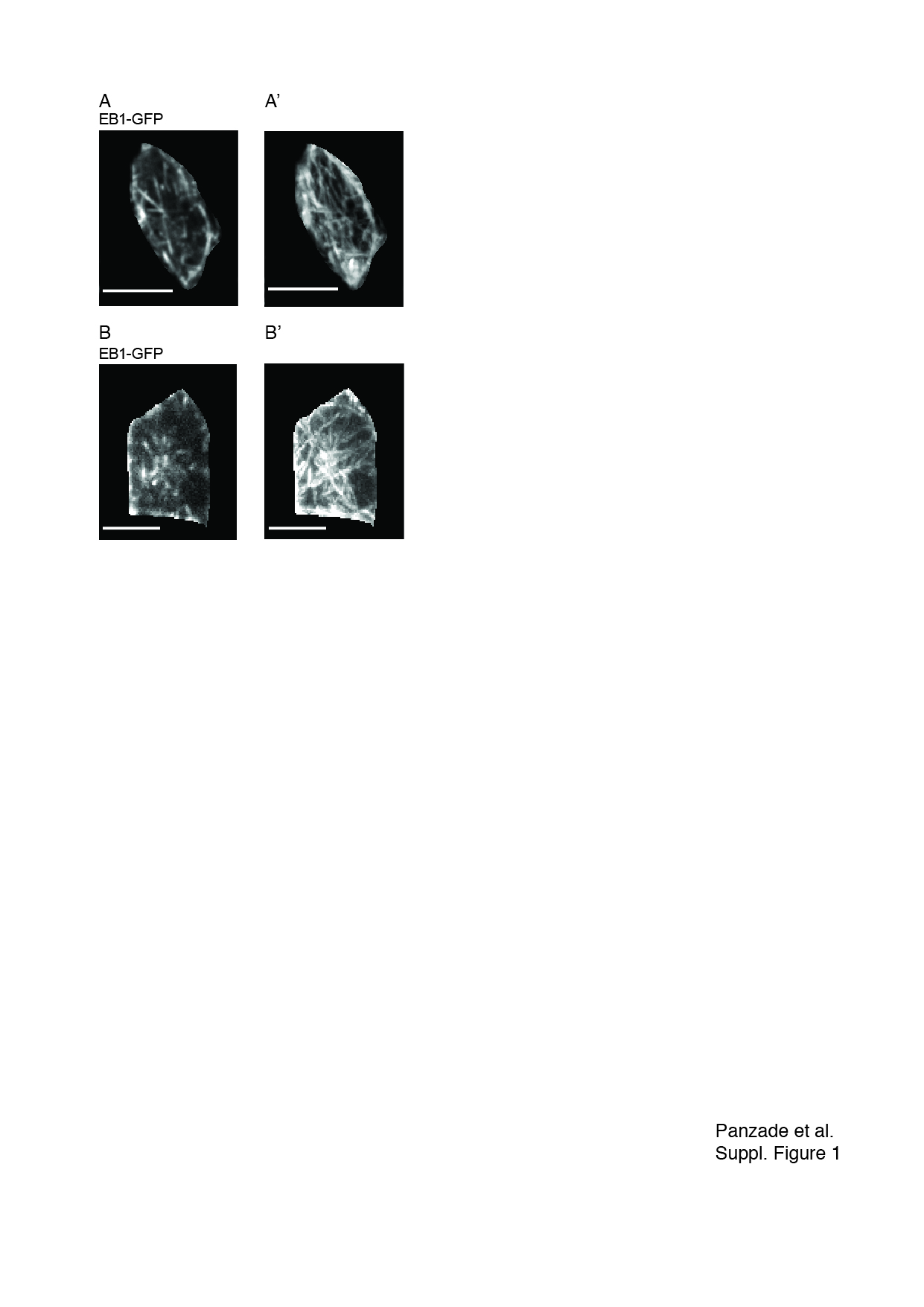

Supplement: Supplementary Figure 1 — Non-centrosomal microtubules in histoblast are aligned along the axis of migration (dorso-ventral axis). (A,B) A representative cell showing EB1 tagged with GFP in wild-type (A) and patroniney05252 mutant histoblast (B). (A’,B’) A maximum intensity projection of an EB1-EGFP time-lapse sequence. Developmental stage (A–B’): 25–26 h APF. Scale bars, 5 μm. [file Image_1.JPEG]

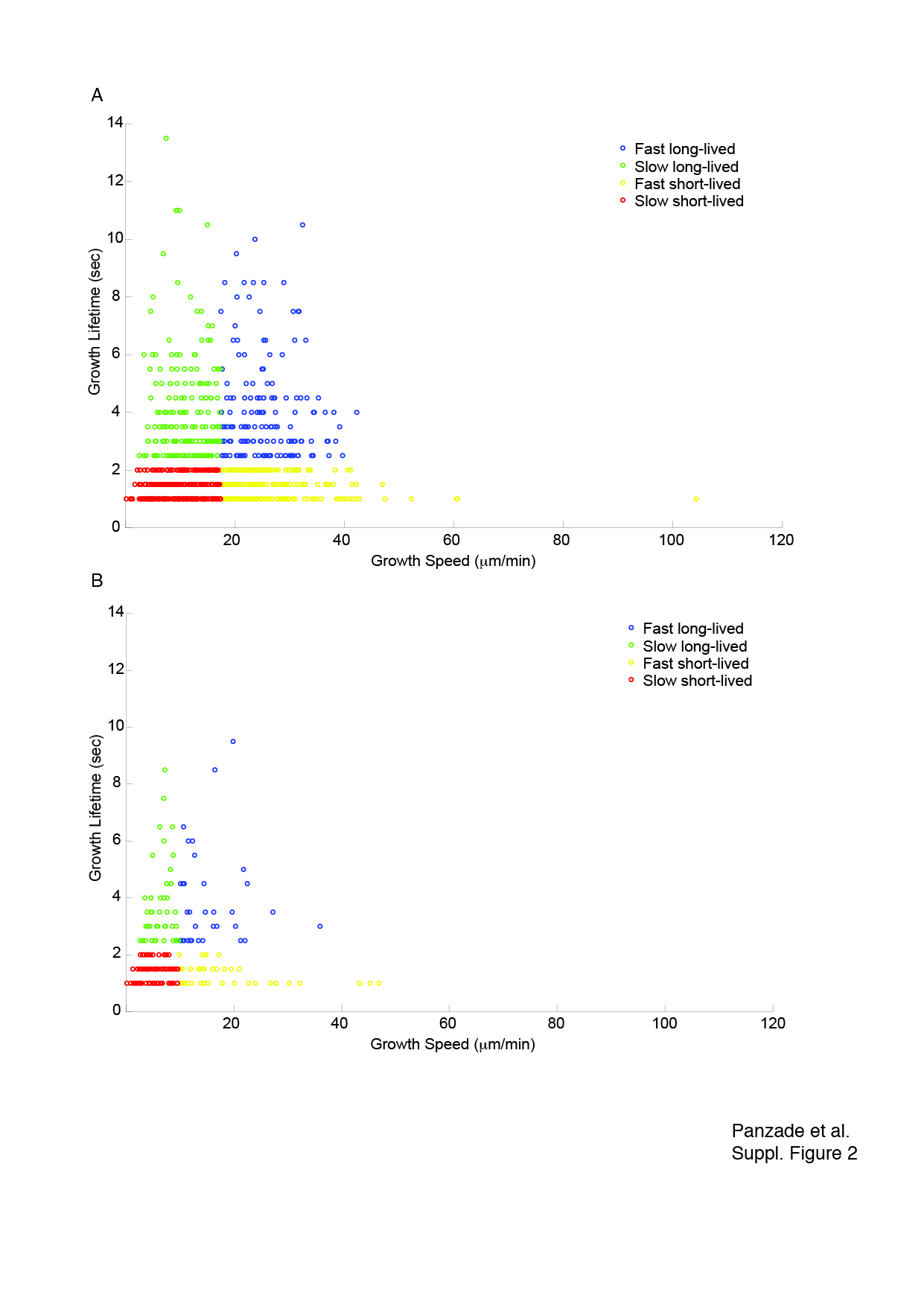

Supplement: Supplementary Figure 2 — Analysis of four distinct dynamic subpopulations in wild-type and patroniney05252 mutant histoblasts. (A) Quadrant scatter plot showing the classification of all detected microtubule growth tracks according to their growth speed and life time in the representative (A) wild-type cell (n) = 750 and (B) patroniney05252 mutant cell (n) = 196. Each point represents one microtubule. Microtubule subpopulations are color coded as, fast and short-lived (yellow), fast and long-lived (blue), slow and short-lived (red), and slow and long-lived (green). [file Image_2.JPEG]

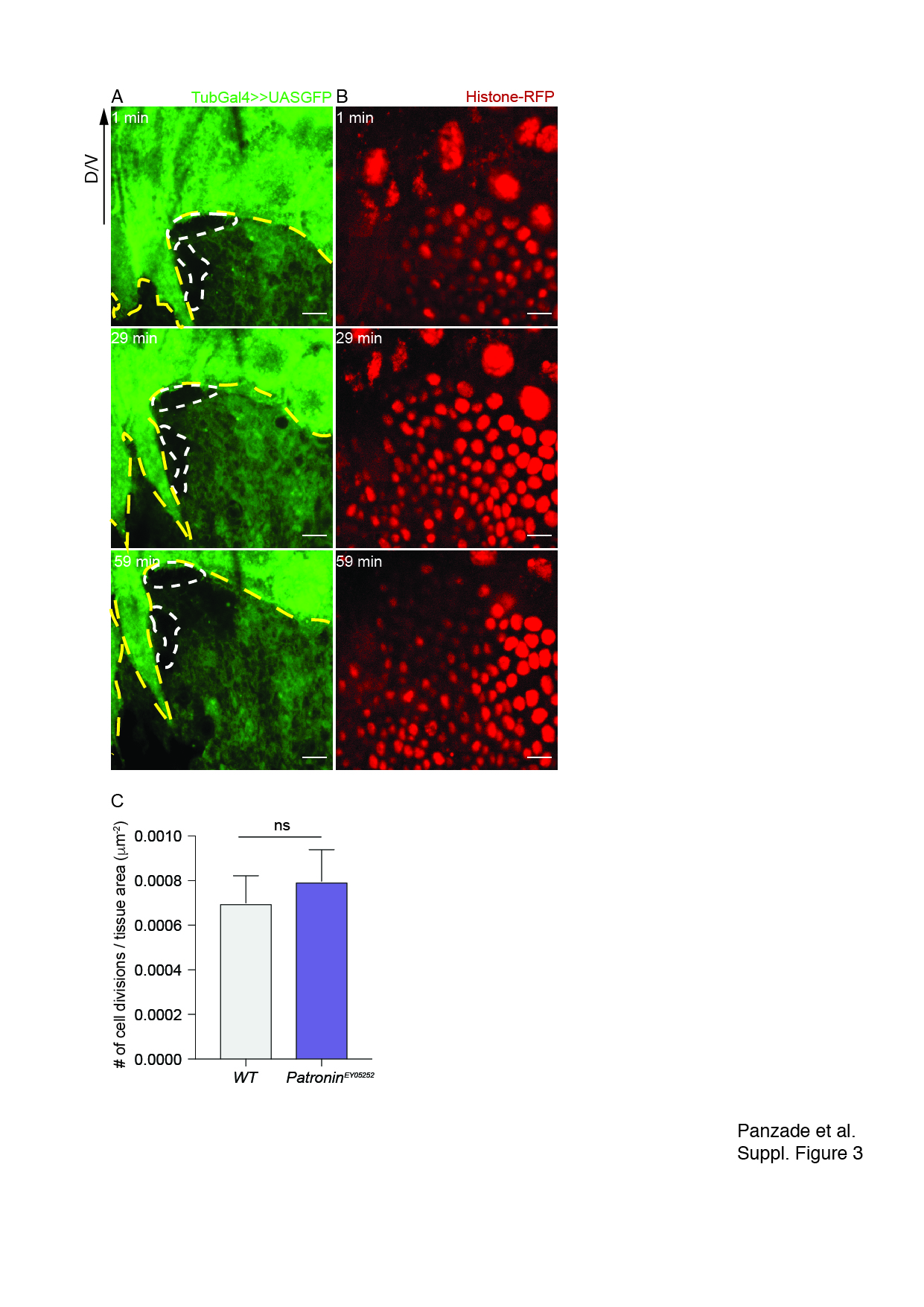

Supplement: Supplementary Figure 3 — Histoblast migration in patroniney05252 mutant flies. (A,B) Live fluorescence images of migrating histoblast in segment A3. The patroniney05252 mutant cells are labeled with the GFP (A) and the nuclei are labeled with the Histone-mRFP (B). A yellow dashed line marks the border between histoblasts and LECs and a white dashed line marks the wild type cells. Panels (A–B) represent snapshots of histoblast from 0 to 59 min during cell migration. Scale bars (A,B), 10 μm. (C) Quantification of the cell divisions per square micrometer in wild type cells and patroniney05252 mutant cells. Bar plot is presented as mean ± SEM. Mann–Whitney U test, n.s., p = 0.6308, number of cell divisions (n) = 90, and pupae (N) = 3 for wild-type cells and number of cell divisions (n) = 146 and pupae (N) = 4 for patroniney05252 mutant tissue. [file Image_3.JPEG]

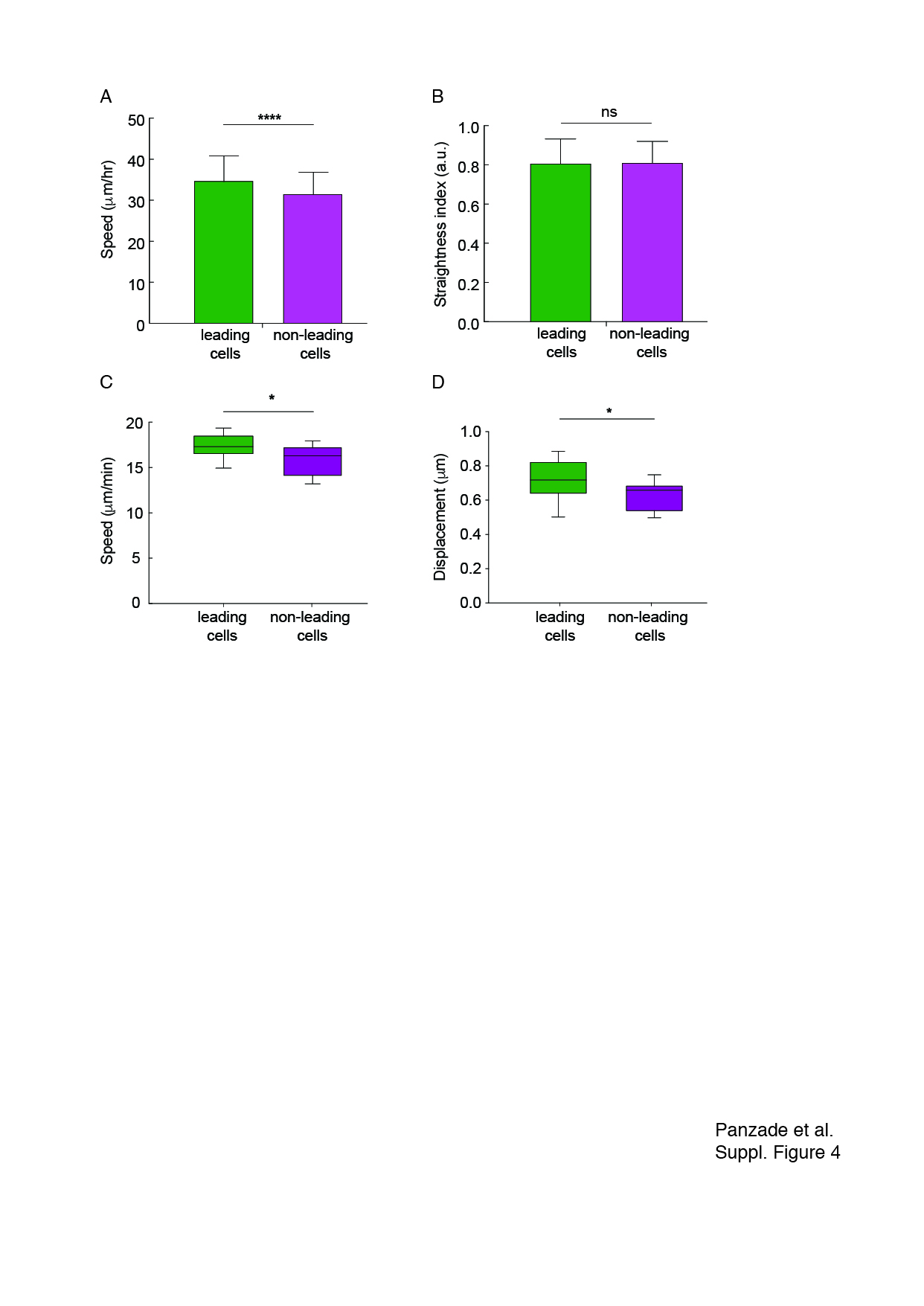

Supplement: Supplementary Figure 4 — Leading and non-leading histoblast migrate same. Quantification of the speeds (A) and the straightness index (B) of leading (green) and non-leading epithelial cells (magenta). (A,B). Bar plots are presented as mean ± SEM. Statistics (A,B), Mann–Whitney U test, n.s., no significant difference; and p < 0.0001 (****). Number of leading cells (n) = 279, and pupae (N) = 3 and non-leading cells (n) = 1,392 and pupae (N) = 3. (C, D) Quantification of microtubule growth speed (C) and displacement length (D) in leading cells (green) and non-leading cells (magenta). Statistics (C,D), Mann–Whitney U test (speed) p = 0.0241 and (displacement length) p < 0.0444. The number of leading cells for (C,D) (n) = 14 and pupae (N) = 3 and non-leading cells (n) = 14 and pupae (N) = 3. Developmental stage (A–B): 26–30 h APF, (C–D): 25–26 h APF. [file Image_4.JPEG]

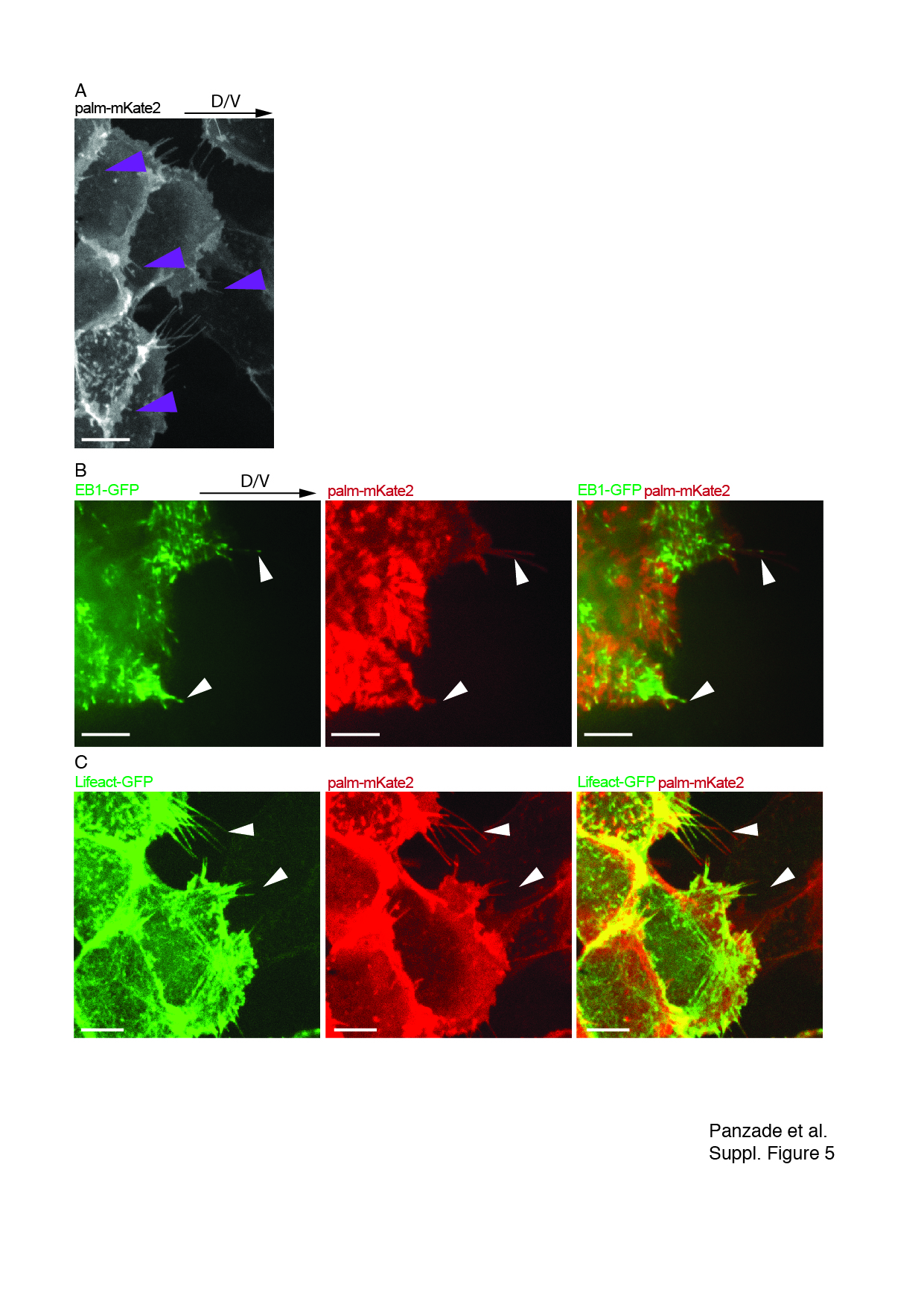

Supplement: Supplementary Figure 5 — Histoblasts protrusions are enriched with actin and microtubules. (A) A representative confocal image showing migrating histoblast expressing membrane marker palm-mKate2. White arrowheads indicate protrusions in non-leading cells. (B,C) Cell protrusions in histoblasts are labeled with the membrane marker palm-mKate2 and microtubules with the plus-end marker EB1-GFP (B) and in panel (C) LifeAct-GFP marks actin. White arrowheads indicate the presence of microtubules (B) and actin (C) in protrusions. Developmental stage (A–C): 25–26 h APF. Scale bars (A–C), 5 μm. [file Image_5.JPEG]
